# Visualization of plant viral suppressor silencing activity in intact leaf lamina by quantitative fluorescent imaging

**DOI:** 10.1186/1746-4811-7-25

**Published:** 2011-08-03

**Authors:** Dirk Stephan, Coba Slabber, Gavin George, Victor Ninov, Kevin P Francis, Johan T Burger

**Affiliations:** 1 Department of Genetics, Stellenbosch University, Private Bag X1, 7602 Matieland, South Africa; 2 Caliper Life Sciences, 2061 Challenger Drive, Alameda, CA 94501, USA

## Abstract

**Background:**

Transient expression of proteins in plants has become a favoured method over the production of stably transformed plants because, in addition to enabling high protein yields, it is both fast and easy to apply. An enhancement of transient protein expression can be achieved by plant virus-encoded RNA silencing suppressor proteins. Since viral suppressor proteins differ in their efficiency to enhance transient protein expression in plants, we developed a whole-leaf green fluorescent protein (GFP)-based imaging assay to quantitatively assess suppressor protein activity.

**Results:**

In a transient GFP-expression assay using wild-type and GFP-transgenic *N. benthamiana*, addition of the plant viral suppressors Beet mild yellowing virus (BMYV-IPP) P0 or Plum pox virus (PPV) HC-Pro was shown to increase fluorescent protein expression 3-4-fold, 7 days post inoculation (dpi) when compared to control plants. In contrast, in agroinfiltrated patches without suppressor activity, near complete silencing of the GFP transgene was observed in the transgenic *N. benthamiana *at 21 dpi. Both co-infiltrated suppressors significantly enhanced GFP expression over time, with HC-Pro co-infiltrations leading to higher short term GFP fluorescence (at 7 dpi) and P0 giving higher long term GFP fluorescence (at 21 dpi). Additionally, in contrast to HC-Pro co-infiltrations, an area of complete GFP silencing was observed at the edge of P0 co-infiltrated areas.

**Conclusions:**

Fluorescence imaging of whole intact leaves proved to be an easy and effective method for spatially and quantitatively observing viral suppressor efficiency in plants. This suppressor assay demonstrates that plant viral suppressors greatly enhanced transient GFP expression, with P0 showing a more prolonged suppressor activity over time than HC-Pro. Both suppressors could prove to be ideal candidates for enhancing target protein expression in plants.

## Background

In recent years the transient expression of proteins in plants has become a favoured procedure over the generation of stably transformed transgenic plants to achieve high levels of protein expression. In contrast to the time-consuming procedure involved in engineering transgenic plants, transient expression methods are more convenient and allow high level protein production in as little as a few days [1,2]. Transient production of proteins is mediated by the introduction of an expression construct into plants which leads to a strong increase in protein synthesis [3]. For high level protein expression, these constructs are preferably under control of a constitutive promoter, e.g. Cauliflower mosaic virus (CaMV) 35S [4], or the protein of interest is included into a modified plant viral expression vector [1,5]. Whatever expression system is used, a normal prerequisite is the testing of such constructs for their expression efficiency in specific host plants, mainly *Nicotiana *ssp., known to give high protein yields [6]. Such efficiency tests often include easy to detect and quantifiable marker proteins. One of these proteins, which does not require any substrate or co-factors, is the green fluorescent protein (GFP), originally isolated from the jellyfish *Aquorea victoria *[7-9].

Green fluorescent protein is widely used as a quantitative marker in plants and other organisms. Quantification of GFP expression in plants requires either anti-GFP antibody for immunological assays or a device able to detect and measure GFP fluorescence [10]. Depending on the experimental layout, a number of different GFP imaging devices and methodologies are currently used, from conventional hand-held UV lamps, through confocal laser-scanning microscopes, some of which also allow for quantitative GFP expression analysis [11-13]. Detection and quantification of GFP is often hampered by auto-fluorescence of plant tissues, which is mainly due to chlorophyll [14]. However, interference by auto-fluorescence of plant tissues can often be reduced or eliminated by specific optical filters [15].

With plant expression systems, high levels of protein synthesis are often impeded by transcriptional or post-transcriptional gene silencing (PTGS) mechanism of the plant [16] leading to a rapid decline in protein yield after the plant's RNA silencing mechanism is triggered. This mechanism is a defence response against external nucleic acids, e.g. plant viruses or artificially introduced transient expression constructs [17]. RNA silencing leads to a sequence specific degradation of RNA and subsequently to a reduction of gene product. Most known plant viruses have been found to counteract the RNA silencing mechanism in plants by encoding silencing suppressor proteins [18]. It has been shown that most plant virus silencing suppressors operate by modifying the accumulation and/or activity of short RNAs, which are a regulatory element in the RNA silencing mechanism [18]. In transient expression assays the silencing effect can be reduced by co-expressed plant viral silencing suppressors [1,19-25], which interfere with the RNA silencing machinery and lead to an increase in transiently expressed proteins [3,26].

We report here the differences in efficiency of two plant viral suppressors, Beet mild yellowing virus P0 (BMYV-P0) and Plum pox virus HC-Pro (PPV-HC-Pro), using a novel whole leaf fluorescent imaging methodology, and demonstrate that over time both viral suppressor proteins can significantly effect GFP expression for up to 21 days post-agroinfiltration. The HC-Pro of potyviruses is an extensively studied viral protein and its suppressor activity has been demonstrated [27,28]. However, variation in P0 suppressor activity between different isolates of the polerovirus BMYV has been shown only recently [29]. We further investigated P0 suppressor activity using an additional BMYV isolate (BMYV-IPP) of which the only infectious BMYV full-length clone is available [30].

For the suppression assays we used an imaging system routinely used for in vivo animal imaging (IVIS^® ^Lumina II, Caliper Life Sciences), demonstrating its equal utility for monitoring gene expression in plants. The system allowed us to quantitatively monitor GFP intensity spatially over the surface of plant leaves and effectively show plant virus suppressor activity.

## Results

### Quantitative analysis of transgene and transient GFP expression from intact leaves

**Figure 1:**
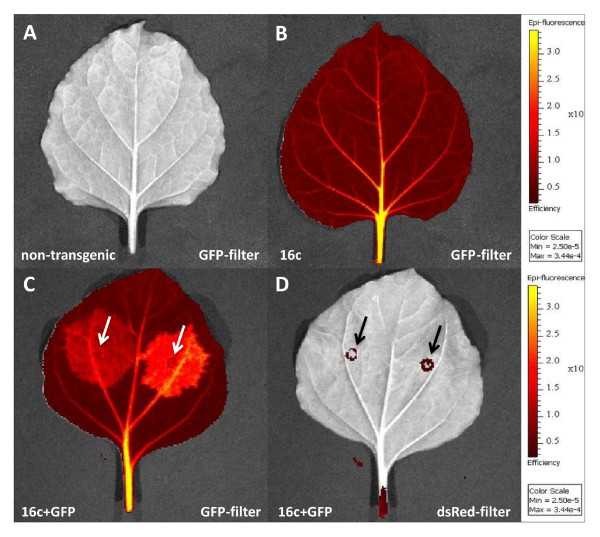
**Epi-fluorescence emission images of GFP-transgenic and non-transgenic *N. benthamiana *leaf samples**. Overlay capture of a plain black/white photograph and the overlaid efficiency image of complete leaves from non-transgenic (A) and GFP-transgenic (line 16c, B) plants using the GFP filter settings of the imaging system. Arrows indicate detected auto fluorescence of necrotic tissue at the onset of the syringe on the same leaf from a GFP-transgenic plant agroinoculated with a GFP-expressing construct using GFP (C) or dsRed (D) filter settings.

In order to verify whether our imaging system was suitable to detect GFP expression from plant tissue, leaves of GFP-transgenic and non-transgenic *N. benthamiana *plants of the same age were harvested and observed simultaneously side-by-side. The transgenic *N. benthamiana *line 16c contains a single copy of a transgene encoding GFP. These plants accumulate high levels of GFP and their leaves and stems fluoresce green under UV illumination. Through selection of a GFP-filter set, transgenic and non-transgenic leaf samples could easily be discriminated. Using the standard settings of the system, non-transgenic leaves showed only background emissions. In contrast, leaves of transgenic plants showed GFP-derived fluorescence throughout the entire leaf area (Figure 1A+B). As one would observe with a conventional hand-held UV lamp, higher GFP-fluorescence could be detected in petioles, leaf midribs and leaf veins, as auto-fluorescence in these areas of the plant is reduced due to a lower content of competing chlorophyll compared to interveinal areas. To allow a quantitative analysis of GFP expression, the measurements in the imaging system were normalized by using an instrument-specific reference image. The data is therefore not displayed as photon counts per area, but as average efficiency. The average efficiency has no units and represents the ratio of emitted to incident light. In GFP-transgenic leaf tissues the average efficiency was about 20 times higher than in non-transgenic control samples.

In order to detect plant viral silencing suppressor activity, leaves of GFP-transgenic plants were infiltrated with recombinant *Agrobacterium-*suspensions. The infiltration procedure made use of a syringe without needle, which inevitably leads to damage of leaf tissue. Autofluorescence of damaged leaf tissue in the infiltrated areas can interfere with accurate detection and quantification of GFP fluorescence [31]. Transgenic leaves infiltrated with *Agrobacterium- *suspensions, which allow for transient GFP expression (pBinGFP) were therefore examined in the system using GFP- and dsRed filter settings. Whereas autofluorescence of damaged leaf tissue will be detected by both filter settings, the dsRed filter covers wavelengths outside that of GFP and will not detect GFP fluorescence but only autofluorescence of damaged leaf tissue. Using the dsRed filter settings no transgene derived or transiently expressed GFP fluorescence was detected and only at the attaching point of the syringe used for infiltration, autofluorescence caused by necrotic plant tissue was observed (Figure 1C+D).

**Figure 2:**
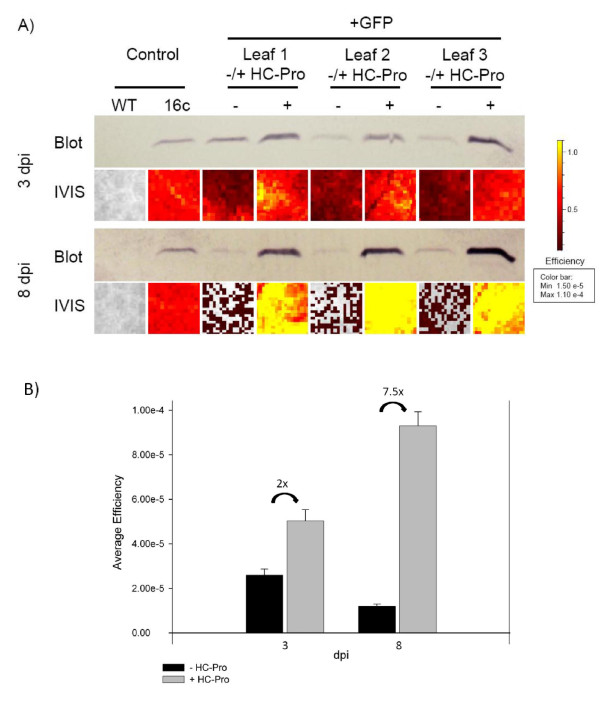
**Comparison of GFP expression detectable by immunoblots and the imaging system**. (A) Identical leaf areas were used to detect and directly compare transient GFP expression in non-transgenic *N. benthamiana *by immunoblots and the imaging system (IVIS) at 3 dpi (top) and 8 dpi (bottom), respectively. The control samples in the first two lanes show GFP detected in non-transgenic (WT) and GFP-transgenic *N. benthamiana *(16c). For each time point, three leaf samples agroinoculated on the same leaf with pBinGFP/pBin (-) and pBinGFP/pBinHCPro (+) are shown. Below each immunoblot sample the magnified pixelated leaf region of the identical sample as measured by the imaging system is shown indicating the average efficiency of GFP expression. Grey pixels indicate complete silencing of the transiently expressed GFP. (B) The average efficiency of GFP expression at 3 (n = 8) and 8 dpi (n = 9) in pBinGFP/pBin (-HC-Pro) and pBinGFP/pBinHCPro (+HC-Pro) agroinfiltrated leaf patches of non-transgenic *N. benthamiana *as measured in the imaging system. The average x-fold increases between patches with and without HC-Pro are indicated.

As the standard GFP-filter of the imaging system allowed a detection of GFP-expression, we further determined if the imaging system allows for an accurate quantification of GFP-expression in intact leaf tissue. For that, transient GFP-expression was measured 3 and 8 dpi in wild-type non-transgenic *N. benthamiana *leaves which were agroinfiltrated with a mixture of recombinant *Agrobacterium *harbouring binary CaMV 35S promoter driven expression constructs expressing GFP (pBinGFP) or the plant viral suppressor PPV HC-Pro (pBinHCPro). As a control, an empty expression construct (pBin) was included. Agroinfiltration of pBinHCPro/pBinGFP and pBin/pBinGFP mixtures were done on either side of the leaf midrib to allow GFP quantification in the same leaf. The GFP-expression of identical leaf tissues was measured first in the imaging system and then after protein extraction compared to detectable GFP by immunoblot analysis (Figure 2A). In agroinfiltrated patches, the average efficiency of GFP expression was already 2 times higher at 3 dpi and 7.5 times higher at 8 dpi in patches co-infiltrated with PPV HC-Pro compared to patches without HC-Pro (Figure 2B). Moreover, the amount of detectable GFP increased substantially in PPV HC-Pro co-infiltrated patches between 3 and 8 dpi, whereas the amount of detectable GFP in patches without suppressor clearly decreased. The quantitative results obtained by the imaging system were verified by quantitative immunoblot analysis of detectable GFP in the respective agroinfiltrated patches. As detected by the imaging system, the same clear difference of detectable GFP in patches with or without suppressor activity at both time points could be observed by immunoblot analysis (Figure 2A).

### Enhancement of GFP expression by PPV HC-Pro in GFP-transgenic *N. benthamiana *over time

**Figure 3:**
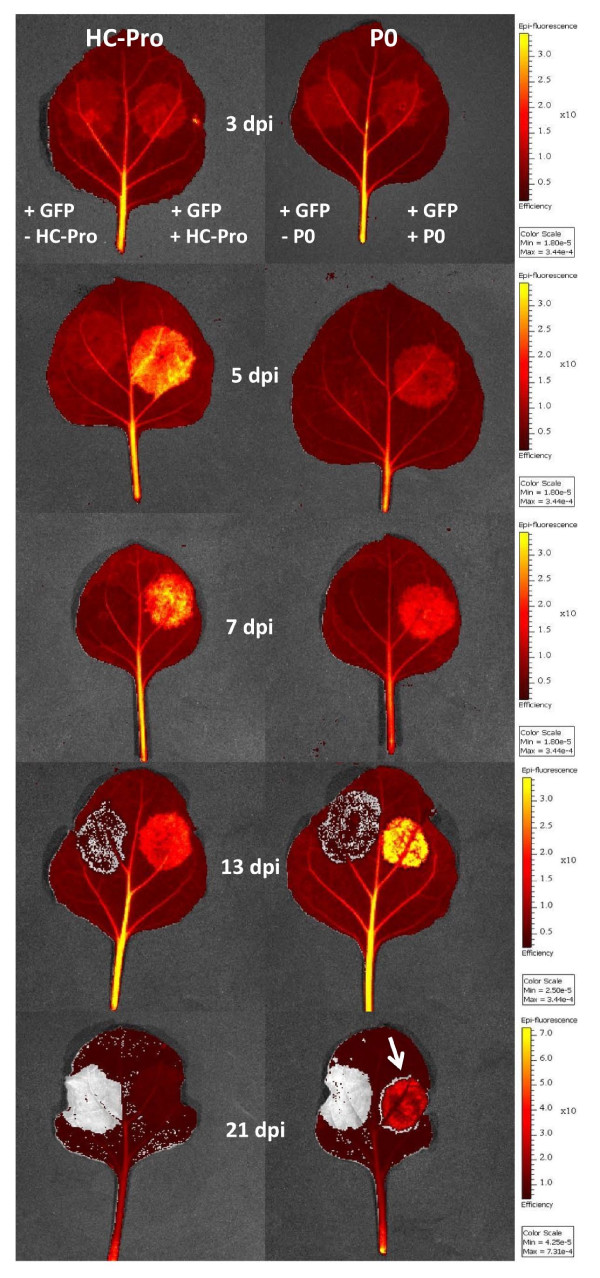
**Comparison of PPV HC-Pro and BMYV-IPP P0 suppressor efficiency**. Overlay capture of a plain black/white photograph and the overlaid efficiency image from GFP-transgenic leaf samples agroinoculated with pBinGFP/pBin (control, left side of leaf midrib) and pBinGFP/pBinHCPro or pBinGFP/pBinP0, respectively (right side of leaf midrib). Samples are taken on 3, 5, 7, 13 and 21 dpi. Colour bars show the average efficiency and min. and max. values between different time points are different but always identical for samples at the same time point. The white arrow indicates an area of completely GFP-silenced tissue (silencing ring) characteristic for P0 suppressor activity.

Given that the imaging system proved capable to accurately assess GFP fluorescence from plant tissue, we quantitatively determined the efficiency of the plant viral suppressor PPV HC-Pro to enhance GFP fluorescence in the *Agrobacterium*-based co-infiltration assay over a longer period of time. Leaves of GFP-transgenic *N. benthamiana *were agroinfiltrated with pBinHCPro/pBinGFP and pBin/pBinGFP mixtures on either side of the leaf midrib (Figure 3). The GFP-transgenic *N. benthamiana *16c was chosen as not only it allows monitoring of the RNA silencing suppression effect on the transiently expressed GFP (pBinGFP) but also on the stable transgenic GFP-expression. Detection and quantification of GFP fluorescence of the agroinfiltrated leaves was done at 3, 5, 7, 13 and 21 dpi in the imaging system.

At 3 dpi, transient expression of GFP could be observed in the infiltrated patches. This was detectable by a higher average efficiency in infiltrated patches compared to the surrounding non-infiltrated tissues. There were no significant differences in average efficiency of GFP expression between pBinHCPro/pBinGFP and pBin/pBinGFP infiltrated tissues. At 5 dpi, there was already a significant difference in average efficiency between the two variants with pBinHCPro/pBinGFP infiltrated tissues showing a two times higher GFP- fluorescence than the control infiltrated patches. At 7 dpi agroinfiltrated patches with HC-Pro expression showed on average a 4.5-fold higher GFP fluorescence than patches without suppressor activity on the same leaf (Figure 3). The GFP expression in the patches without co-agroinfiltrated HC-Pro was at that time point already slightly below that of the surrounding non-infiltrated plant tissue (Figure 3) indicating a successful silencing effect on the transgene and transiently expressed GFP, respectively. At 13 dpi the average GFP signal decreased by 39% and 34% in patches without and with co-infiltrated HC-Pro, respectively. However, HC-Pro co-infiltration still showed to a 5-fold increase in GFP signal. In patches without suppressor activity, areas were detected in which transient and transgene GFP expression was not detectable at all (Figure 3, interspersed grey areas without color coded pixels). In all agroinfiltrated patches without suppressor activity, GFP intensity was lower than in the surrounding GFP-transgenic tissues. At 21 dpi the GFP intensity decreased further in agroinfiltrated patches without and with suppressor activity, respectively, but HC-Pro agroinfiltrated patches still showed a 3-fold higher GFP signal than the control (Figure 3). Similar and more obvious to the observations on 13 dpi, in patches without suppressor activity the GFP expression seemed to be completely silenced or greatly reduced below normal GFP-transgene level expression at 21 dpi (Figure 3). Except for 3 dpi, at all other time points measured patches co-agroinfiltrated with PPV HC-Pro always showed a significant higher average efficiency in GFP expression than the control.

Small necrotic lesions around the periphery of the attaching point of the syringe for agroinfiltration were present in both, pBinHCPro/pBinGFP and pBin/pBinGFP infiltrated areas. No differences in autofluorescence as detected by dsRed filter settings were observed between both variants.

### Enhancement of transient GFP expression by BMYV P0 in GFP-transgenic *N. benthamiana *over time

In parallel to the measurements using PPV HC-Pro as a suppressor the efficiency of BMYV-IPP P0 to enhance GFP expression in a transient assay was tested.

**Figure 4:**
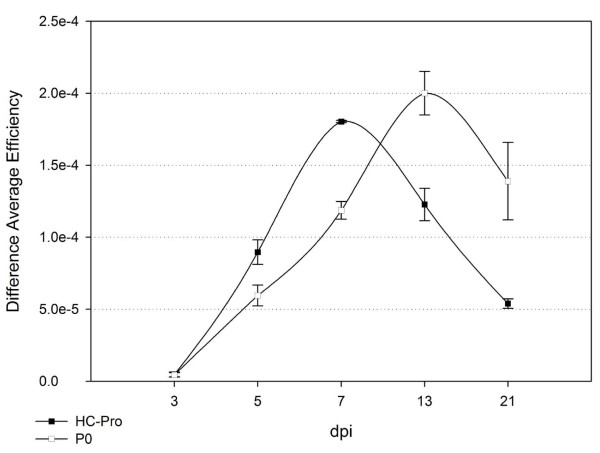
**PPV HC-Pro and BMYV-IPP P0 suppressor activity at 3, 5, 7, 13 and 21 dpi**. Diagram showing the mean difference in average efficiency of control (pBinGFP/pBin) and pBinGFP/pBinHCPro (black square) or pBinGFP/pBin P0 (white square) agroinoculated GFP-transgenic leaf patches at 3, 5, 7, 13 and 21 dpi. Differences were calculated for each individual leaf and then combined as mean difference of average efficiency. The standard error is indicated and the number of samples (n) per time point was as follows (HC-Pro/P0): 3 dpi (n = 7/14), 5 dpi (n = 13/17), 7 dpi (18/18), 13 dpi (8/8) and 21 dpi (9/6).

Similar to what was observed for PPV HC-Pro, the average efficiency of GFP fluorescence in pBinP0/pBinGFP and pBin/pBinGFP agroinfiltrated tissues was not significantly different at 3 dpi. However, at 5 dpi the GFP-fluorescence in tissues with P0 activity was 1.3-fold higher than in control tissues. Similar to what was observed for HC-Pro, the GFP signal detected in agroinfiltrated patches was on average 3-fold higher in patches co-infiltrated with P0 compared to controls at 7 dpi (Figure 4). In patches without P0 activity the GFP signal was below that of the surrounding GFP-transgenic leaf tissue and in some areas not detectable at all (Figure 4). At 13 dpi the overall GFP-intensity in all patches decreased by 42% and 26% without or with P0 co-infiltration, respectively, but was still 8-fold higher in P0 co-infiltrated areas. Areas without detectable GFP-expression were seen to increase in control infiltrations without suppressor activity (Figure 4). At 21 dpi GFP-intensity in P0 agroinfiltrations was still 6-fold higher than in patches without suppressor activity, where in the later case some patches did not show any GFP expression at all. Even if the general GFP-intensity between 13-21 dpi decreased in P0 infiltrated patches it was on average still 50% higher than in comparable HC-Pro agroinfiltrations. In contrast to HC-Pro, P0 agroinfiltrated patches showed a great reduction of GFP intensity bordering the agroinfiltrated tissue. This silenced ring could easily be detected using the imaging system as a grey circle surrounding the infiltrated patch (Figure 4, 21 dpi).

## Discussion

The IVIS^® ^Lumina II optical imaging system, used predominantly for non-invasive biophotonic imaging in rodent animal models [32,33], proved to be an ideal tool for the quantitative analysis and spatial distribution of GFP expression in leaves of *N. benthamiana *plants. Utilizing the standard GFP filter settings and software of the system we were able to greatly reduce the background fluorescence from chlorophyll in the plant tissue, which has been reported to be especially problematic when imaging GFP reporters [14]. The imaging system allowed us to rapidly analyse a high number of whole leaf samples under identical conditions in a quantitative manner and accurately assess plant viral suppressors of silencing. The quantitative analysis using the imaging system was successfully confirmed by immunoblot analysis.

Routinely, analysis of plant viral suppressor efficiency is done via agroinfiltration assays that utilize GFP as a visual marker [26]. The simultaneous agroinfiltration of a GFP and suppressor-expressing construct in comparison to infiltrations without suppressor is often the first procedure in establishing a suppressor activity of a specific protein. In these assays, the suppressor activity of a given protein or molecule will ideally delay the plant-derived silencing effect which can be monitored by enhancement and elongation of transiently expressed markers like GFP. Nevertheless, these assays tend to be more qualitative rather than quantitative [3]. Depending on the experimental layout, different GFP detection methods are currently used, including a conventional hand-held UV lamp, fluorometers, fluorescent immunoassays, laser-induced fluorescent spectroscopy, fluorescence microscopy or confocal laser-scanning microscopy, some of which allow for quantitative GFP expression analysis [10-13]. Our results show that whole lamina imaging in an optical imaging system such as the IVIS^® ^Lumina II allows an accurate assessment of GFP-expression to be determined in plants, and that this methodology is highly applicable to the widely used agroinfiltration assay. Expression of GFP is accurately measured by the imaging system as was shown when comparing to GFP detection by immunoblot analysis.

Other recently described procedures for the quantification of suppressor activity are based on particle bombardment of bean cotyledon tissue [3,22]. However, the latter method does not allow spatial reporter expression to be assessed, such as the development of a silencing ring around infiltrated patches. Additionally, keeping in mind that whole plant agroinfiltration procedure might be combined with a suppressor to increase and extend foreign target protein expression [25], particle bombardment assays might not reflect the same suppressor activity as in agroinfiltrations.

Our results are in line with earlier reports and showed that without co-agroinfiltrated suppressors, GFP-expression in transgenic and non-transgenic *N. benthamiana *declines rapidly after a peak at 3-4 dpi. This was detected in GFP-transgenic and non-transgenic plants. Moreover, in our assay GFP silencing continued until GFP expression can no longer be detected (transient and transgenic) at 21 dpi in our assay. We did not detect transient GFP-expression, with or without added suppressor, after 24 hours [data not shown] as it was described earlier using a particle bombardment assay [3,22]. This difference might be due to the delivery procedure itself or the plant species or tissue used.

When comparing PPV HC-Pro suppression activity between GFP-transgenic and non-transgenic *N. benthamiana *we observed a similar pattern in increase or decrease of GFP expression over time. Nevertheless, a doubling of GFP expression in patches with HC-Pro activity when compared to the control on the same leaf was reached in non-transgenic plants two days earlier than in GFP-transgenic plants. Similarly, the GFP expression status with or without HC-Pro detected at 8 dpi in non-transgenic plants was comparable to the one in GFP-transgenic plants on 13 dpi. This might simply reflect the constitutive GFP expression in transgenic plants which will mask to some degree the effect of HC-Pro suppression activity on transiently expressed GFP.

Similar to what was described for Beet western yellows virus-derived P0 and Potato virus Y-derived HC-Pro [34], at 5 dpi GFP-expression in patches co-infiltrated with PPV HC-Pro or BMYV-IPP P0 suppressors was greatly enhanced in contrast to patches without suppressor. This enhancement by PPV HC-Pro and BMYV-IPP P0 was detectable until 21 dpi (the last time point) with HC-Pro and P0 leading to highest GFP intensities at 7 dpi or 13 dpi, respectively. The marked differences between both PPV HC-Pro and BMYV-IPP P0 suppressors are (i) a higher average GFP-intensity induced by PPV HC-Pro at 7 dpi, (ii) BMYV-IPP P0 leading to a more prolonged suppressor activity than HC-Pro, which may be explained by P0 being more stable or a more efficient suppressor than HC-Pro, as was previously suggested for BWYV P0 [34] and (iii) BMYV P0, similar to BWYV P0 and in contrast to HC-Pro, does not suppress cell-to-cell spread of the silencing signal from agroinfiltrated tissues, indicated by the observed silencing ring around agroinfiltrated tissues [35]. These observations reflect the different modes of action reported for HC-Pro and P0 in suppression of RNA silencing: whereas HC-Pro binds small RNAs and therefore impedes their loading onto the RISC complex and cell-to-cell spread, P0 mediates ARGONAUTE1 degradation, a key component of the RISC complex itself, and therefore does not interfere with cell-to-cell movement of the silencing signal [36].

An interesting observation is the increasing standard derivation (Figure 4) of the difference in average efficiency between 7 and 21 dpi in samples co-agroinfiltrated with the suppressor P0 which was not detected in that instance for HC-Pro or between controls. The difference in average efficiency reflects the comparison of measurements between the control and suppressor on individual leaves. Even if not statistically significant, the increasing variation of P0 suppressor efficiency to the end of the experiment might reflect a higher dependence of P0 suppressor activity on the individual status of a leaf or plant.

This is the first combined qualitative and quantitative description of BMYV-IPP P0 and PPV HC-Pro suppressor activity in plants, adding the P0 of the only infectious BMYV full-length cDNA clone [30] to the list of effective beet- infecting poleroviral suppressors [29].

## Conclusions

Using quantitative fluorescent imaging of intact leaf lamina, we were able to accurately assess plant viral suppressor activity both spatially and longitudinally in GFP-transgenic and non-transgenic *N. benthamiana*. This imaging approach should provide an excellent methodology to analyse other viral suppressors and compounds for their ability to enhance protein expression in plants. The procedure enables a large number of samples to be rapidly analysed and opens up the possibility of allowing automated measurements over time, which would be appealing for many areas of plant research. Furthermore, since the imaging system was designed to allow multiple optical reporters, fluorescent and bioluminescent, to be visualized simultaneously, several different dynamic/molecular events could be recorded together. Both, PPV HC-Pro and BMYV-IPP P0 were shown to be useful to enhance and extend GFP-expression in *N. benthamiana *making them possible candidates for co-agroinfiltrations in small and large-scale plant expression systems. Plum pox virus HC-Pro might be used in expression systems where the target protein needs to be harvested early after infiltration, e.g. if it is not stable in plants, and as BMYV P0 seems to have a prolonged suppressor activity it might be co-agroinfiltrated with any target protein construct to allow high protein yields.

## Methods

### Plasmid constructs

Binary plasmids pBin61S (pBin) and the PPV HC-Pro containing pBinPPVHC-Pro were already described earlier [18,28]. For the construction of pBinBMYVP0, the BMYV-IPP ORF0 coding region was PCR-amplified using the infectious BMYV-IPP full-length cDNA clone [30] as the template. PCR-added restriction enzyme recognition sites (*Bam*HI/*Xba*I) allowed cloning of the P0 coding region between the left and right border sequences of the binary vector containing a CaMV 35S promoter and termination signal.

### Plant material and agroinfiltration

Plants of the constitutively GFP-expressing *N. benthamiana *line 16c [37] were grown in controlled greenhouse conditions at 25°C/16 h day and 20°C/8 h night time conditions. Plants were grown in sand with controlled irrigation, supplemented with nutritional solution. Plasmid constructs were electroporated into *Agrobacterium tumefaciens *strain C58C1. Leaf infiltrations with recombinant bacterial suspensions were mainly done as described earlier [38]. The *Agrobacterium *cultures were resuspended in buffer (100 mM MgCl_2_, 100 mM MES and 100 μM acetosyringone) to a final OD_600 _of 0.5. In the co-infiltration experiments the pBinGFP containing suspension was mixed with either pBin (control), pBinHCPro or pBinP0 in a 1:3 (GFP:suppressor or control) ratio. On each leaf, patches of about 2-3 cm were infiltrated into lower leaf surface with suspensions containing pBinGFP/pBin on one side of the midrib or pBinGFP with one of the suppressor constructs (pBinGFP/pBinHCPro or pBinGFP/pBinP0) on the opposite side of the midrib by using a syringe without needle. Green fluorescent protein transgenic *Nicotiana benthamiana *were agroinfiltrated at a 4-6 leaves stage. Complete leaves of a comparable age were harvested at 1, 3, 5, 7, 13 and 20 dpi and used for image data acquisition and analysis by comparing the two infiltrated patches on each leaf.

### Serological GFP expression analysis

Leaf material from individual agroinoculated patches was excised and used for serological GFP expression analysis. Immunoblot detection of GFP was performed by separating 0.8 μg total leaf protein on a 10% (v/v) denaturing SDS-PAGE gel. Following the electrophoresis, the protein was transferred to a PVDF membrane (Amersham Highbond-P, GE Healthcare) using a semi-dry blotter (Trans-Blot SD, Biorad). GFP protein was then detected using commercial antibodies according to the manufacturer's instructions (Invitrogen, SKU# A-6455).

### Quantitative GFP expression analysis

Quantitative and qualitative analysis of GFP-expression was done by using the IVIS^® ^Lumina II imaging and the Living Image software version 3.0 (Caliper Life Science). The GFP filter sets in the system include an emission filter (515-575 nm), excitation filter (445-490 nm) and a background filter (410-440 nm). The blue-shifted background filter emits light at a shorter wavelength. The system was set-up with the following parameters using the locked GFP- or dsRed filter for the fluorescent image: subject height 0.5 cm, exposure time 0.5 seconds, binning medium, f/stop 2, field of view 12.5 cm and lamp level high. For each sample a black and white photograph was taken using the automatic setting for the exposure time, binning medium and f/stop 16. The infiltrated patches were individually marked as regions of interest (ROI) in the Living Image software. The ROI allows a measurement of the average GFP fluorescence in a defined area. To allow a quantitative analysis, GFP fluorescence was presented as efficiency and not total photon counts per area. The detection of fluorescent signals emitted from a sample depends on the amount of fluorophore present in the sample and the intensity of the incident excitation light. The excitation light incident on the sample stage is not uniform over the field of view as it peaks in the centre of the field of view and declines to the edges. By using the software internal efficiency feature the extinction light will be eliminated as a variable from the measurement by normalizing the data with an instrument specific calibrated reference image. The data presented here is displayed as average efficiency and has no units and represents the ratio of emitted to incident light. The efficiency number for each pixel displays the fraction of fluorescent photons relative to each incident excitation photon and is typically in the range of 10^-2 ^to 10^-9^. In the ROI measurements described here the average efficiency within a ROI is the efficiency per pixel integrated over the ROI area in cm^2^. The ROI measurements were made for the two infiltrated patches per leaf separately. For each leaf, the difference in average efficiency between control and suppressor treatment was determined. Each leaf was photographed with and without GFP filter settings and the software generates an overlay picture, which could be qualitatively analysed (e.g. detection of silencing ring).

### Data analysis

The obtained data was statistically analysed to allow comparisons of measurements on the same leaf (paired t-test) but also between HC-Pro and P0 suppressor of silencing treated plants. An F-test for homogeneity of variances and a t-test with Bonferroni adjustment to control type I error rates with multiple t-tests at a 95% confidence level was done to determine significances between the independent treatments with the two suppressors.

## Competing interests

The authors declare that they have no competing interests.

## Authors' contributions

DS drafted the manuscript, designed the study and carried out agroinoculations of the different constructs with CS, made the measurements in the Ivis Ilumina System and interpreted the data. GG carried out the serological detection of GFP expression. CS, GG, KF and VN helped with the design of the study, the correct interpretation and statistical analysis of the data. JB participated in the design of the study and the interpretation of the results. All authors read and approved the final manuscript.
